# Dynamic distribution of gut microbiota-metabolites during post-weaning longissimus dorsi muscle development in Ningxiang pigs

**DOI:** 10.1128/spectrum.00813-24

**Published:** 2024-08-20

**Authors:** Jiayi Su, Jinxuan Li, Md. Abul Kalam Azad, Wenliang Wang, Zhili Luo, Jing Wang, Jie Yin, Yulong Yin, Bie Tan, Jiashun Chen

**Affiliations:** 1 Animal Nutritional Genome and Germplasm Innovation Research Center, College of Animal Science and Technology, Hunan Agricultural University, Changsha, Hunan, China; 2 CAS Key Laboratory of Agro ecological Processes in Subtropical Region, Institute of Subtropical Agriculture, Changsha, Hunan, China; University of Arkansas Fayetteville, Fayetteville, Arkansas, USA

**Keywords:** dynamic, microbiota, metabolites, Ningxiang pig, intramuscular fat

## Abstract

Ningxiang pigs (NXPs) have a strong ability to deposit fat and intramuscular fat (IMF). However, microbiota-metabolite development and the role in IMF deposition have been rarely reported. Here, we compared the gut microbiota and metabolite profiles and IMF content at 30, 70, 150, 200, and 250 days of age of NXPs. The results revealed that the IMF content in NXPs increased significantly (*P* < 0.05) as the pigs’ age extended. Additionally, the C14:0 content in the longissimus dorsi muscle at 30 and 70 days of age was significantly lower (*P* < 0.05) than that at 150 and 200 days of age. The Shannon index and ACE index showed a pattern of initially increasing and then decreasing. LEfSe analysis revealed that 41 differential bacteria at the genus level were specific to different growth stages, indicating the dominant bacteria’s dynamic changes in the NXPs during different stages of age. Furthermore, we found that there were significant differences in cecal metabolism, the classification of differential metabolites revealed that 15.61% of compounds were fatty acyls, 13.98% were prenol lipids, and 10.57% were steroids and steroid derivatives. Next, the network analysis showed that *Lachnospiraceae-XPB1014-group* was positively related to 4-2-Aminophenyl-2-4-dioxobutanoic-acid, (Z)-3-Octene, 5-Methyl-furaldehyde, Propyl-2-4-decadienoate, which were also positively correlated with the IMF content. Our findings illustrated the dynamic distribution of cecal microbiota and metabolite composition at different growth stages in NXPs and their correlation with IMF deposition. These results provide a valuable insight into optimizing meat quality and overall health in post-weaning NXPs, providing a foundation for enhancement in pork product.

**IMPORTANCE:**

Understanding the dynamic interplay between gut microbiota, metabolites, and intramuscular fat (IMF) deposition in pigs at various growth stages holds significant importance for the pork industry. This research sheds light on how the composition of gut microbiota and metabolites changes throughout the developmental stages of pigs, impacting IMF content in meat. By identifying specific bacterial genera and metabolites associated with IMF deposition, this study offers valuable insights for optimizing meat quality and health in post-weaning pigs. Such knowledge could lead to targeted interventions or management strategies aimed at enhancing pork product quality and overall profitability for producers. Ultimately, this research contributes to advancing our understanding of the complex relationship between gut microbiota, metabolites, and meat quality, offering practical implications for the swine industry.

## INTRODUCTION

As society evolves and people’s living standards rise, the emphasis on meat consumption has shifted from sheer “quantity” to a greater emphasis on “quality” (1). Fatty acids, lipid compositions, and their distributions are intricately linked to meat quality. A particular emphasis is placed on intramuscular fat (IMF) content, commonly referred to as marbling, which plays a crucial role in various meat quality traits. It contributes significantly to sensory properties such as flavor, juiciness, and tenderness (2).

Recent years, numerous Chinese indigenous pig breeds are prized by consumers for their favorable meat quality. Ningxiang pigs (NXPs), a locally adapted fat-type breed from Hunan province of China, have a strong ability to deposit fat and have more generous IMF than meat from foreign commercial pigs (3). Therefore, the intricate dynamics of IMF accumulation in NXPs during post-weaning growth directly influences the quality and market value of pork products derived from this breed (4).

Microbiota and metabolites in the gut play crucial roles in improving the pork quality and lipid metabolism in pigs (5). Pigs have a vital symbiotic relationship with a diverse gastrointestinal microbiota, primarily located in the cecum (6). The gut microbiota, with its dynamic composition and functional capabilities, can modulate the host’s metabolism and energy balance, influencing the storage and utilization of fats within skeletal muscles (7). Moreover, metabolites like lipids, originating from microbial and host metabolism, contribute to the dynamic milieu that influences adipogenesis and lipid accumulation in muscle tissues (8). Previous studies have rarely undertaken a combined analysis of 16S rDNA surveys and metabolomics to investigate IMF deposition in NXPs. Delving into these realms allows us to uncover the profound impact of intricate interactions on IMF deposition in pigs.

In this study, we not only investigated the temporal changes in myofiber properties, fatty acid, IMF of longissimus dorsi (LD), the gut microbiota, and metabolite profiles but also uncovered the intricate associations among them during post-weaning growth in NXPs. Our research aims to contribute valuable insights into optimizing meat quality and overall health in post-weaning NXPs, providing a foundation for enhancement in pork product.

## MATERIALS AND METHODS

### Animals, diets, and sampling

Ninety castrated male NXPs were carefully chosen and given a standardized swine diet in the same environmental and feeding conditions at the Ningxiang Pig Conservation Center in Hunan, China. During the whole period, the pigs were housed in one pigsty with 10 pens. The temperature of the pigsty is maintained at 25°C to 28°C, and the relative humidity was between 55% and 75%. Feed and clean drinking water were provided *ad libitum* throughout the experiment period. The diets were formulated using corn-soybean meal and met or exceeded the nutrient requirements for NXPs recommended by the Nutrient Requirements of Swine in China (NRSC, 2019). Table 1 shows the ingredients used in the diets for NXPs. The pigs were weaned when they were 30 days old and were fed pre-starter diets for 1 week, followed by grower diets for the remainder of the study. Six pigs with similar body weights were selected for sampling at each of the five developmental stages (30, 70, 150, 200, and 250 days of age). After weighing the pigs (12 hours after the last feeding), they were humanely euthanized using general anesthesia (9). The contents of the middle section of the cecum were collected individually and quickly frozen in liquid nitrogen before being stored at −80°C for further analysis of microbiota and metabolome. LD samples were extracted from the carcass, and any visible fat was removed before being vacuum sealed. Some LD samples were preserved in paraformaldehyde for histological staining, while others were frozen at −20°C until they could be analyzed for fatty acids.

**TABLE 1:** Composition and nutrient content of experimental diets (as-fed basis)

Items	Content of 30–50-kg NXP feed (%)	Content of 50–80-kg NXP feed (%)
Ingredients		
Corn	67.80	62.13
Soybean meal	16.50	5.60
Rice bran	10.00	0
Wheat bran	1.75	27.60
Limestone powder	0.90	0.95
Calcium hydrogen phosphate	0.70	0.85
L-Lysine-HCl	0.50	0.52
L-Threonine	0.05	0.05
Salt	0.30	0.30
Vitamin and mineral premixs^*a*^	1.50	2.00
Total	100	100
Calculated nutrients		
Digestible energy (MJ/kg)	13.83	12.23
Crude protein	14.22	12.11
Crude ash	4.86	4.23
Lysine	0.68	0.85
Methionine	0.15	0.17
Methionine + cysteine	0.36	0.35
Threonine	0.32	0.36
Calcium	0.67	0.61
Total phosphorus	0.53	0.55

^*a*^ 30–50-kg NXP feed premix provided the following for per kg of diets: Fe, 90.00 mg; Cu, 4.00 mg; Mn, 3.00 mg; Zn, 65.00 mg; I, 0.15 mg; Se, 0.45 mg; vitamin A, 4,500 IU; vitamin D3, 625 IU; vitamin E, 100.00 IU; vitamin K, 2.80 mg; vitamin B12, 25 μg; vitamin B1, 2.50 mg; vitamin B2, 5.50 mg; vitamin B6, 3.00 mg; D-pantothenic acid, 15.50 mg; niacin, 35.00 mg; biotin, 0.10 mg; folic acid, 0.50 mg; choline, 450.00 mg. 50–80-kg NXP feed premix provided the following for per kg of diets: VA 5060 IU, VD3 3030 IU, VE 40.6 IU, VK3 5.0 mg, VB12 20.0 μg, VB1 2.0 mg, VB2 8.0 mg, pantothenic acid 15.2 mg, niacin 20.3 mg, choline chloride 609 mg, Mn 61.8 mg, Fe 109.2 mg, Zn 104 mg, Cu 22 mg, I 0.3 mg, and Se 0.31 mg.

### Histological staining

The samples of LD muscle were fixed with 4% paraformaldehyde for 1 hour. After fixation, the tissues were treated with water washing, transparency, wax immersion, and embedding at room temperature. A series of 5-µm paraffin-embedded sections were then prepared and stained with hematoxylin and eosin (Sigma-Aldrich, St. Louis, MO, United States) (10).

### Determination of fatty acids and intramuscular fat content of the LD muscle

Approximately, 0.3–0.5-g samples were weighed into 10-mL centrifuge tubes and mixed with 5 mL methanol: chloroform (1:2) solution. The samples were then shaken in a shaker for 1 hour, filtered through quantitative filter paper, and centrifuged with 4 mL of distilled water at 845 g for 5 minutes at 4°C. The supernatants were collected, and the lower layers were drained out with negative pressure in a water bath at 40°C. Next, 1 mL of chromatographic-pure hexane was added to dissolve the oil, and 1 mL of 0.4 mol/L KOH-methanol solution was added and stored for 30 minutes for methylation. After that, 2 mL of deionized water was added. Following esterification, the upper solutions were extracted and the fatty acid contents were measured using gas chromatography (Agilent 7820a, Agilent Technologies, USA). The IMF content was measured using the Soxhlet extraction method (11).

### Analysis of gut microbiota of cecal contents

Microbial DNA was extracted from fecal samples using the E.Z.N.A. soil DNA Kit (Omega Bio-tek, Norcross, GA, U.S.) according to manufacturer’s protocols. The final DNA concentration and purification were determined by a NanoDrop 2000 UV-vis spectrophotometer (Thermo Scientific, Wilmington, USA), and the DNA quality was checked by 1% agarose gel electrophoresis. The V3-V4 hypervariable regions of the bacteria 16S rRNA gene were amplified with primers 338F (5′-ACTCCTACGGGAGGCAGCAG-3′) and 806R (5′-GGACTACHVGGGTWTCTAAT-3′) by thermocycler PCR system (GeneAmp 9700, ABI, USA). The PCR reactions were conducted using the following program: 3 minutes of denaturation at 95°C, 27 cycles of 30 s at 95°C, 30 s for annealing at 55°C, and 45 s for elongation at 72°C and a final extension at 72°C for 10 minutes. PCR reactions were performed in triplicate 20-µL mixture containing 4 µL of 5× FastPfu Buffer, 2 µL of 2.5 mM dNTPs, 0.8 µL of each primer (5 µM), 0.4 µL of FastPfu Polymerase, and 10 ng of template DNA. The resulted PCR products were extracted from a 2% agarose gel and further purified using the AxyPrep DNA Gel Extraction Kit (Axygen Biosciences, Union City, CA, USA) and quantified using QuantiFluor-ST (Promega, USA) according to the manufacturer’s protocol. Purified amplicons were pooled in equimolar and paired-end sequenced (2 × 300) on an Illumina MiSeq platform (Illumina, San Diego, USA) according to the standard protocols by Majorbio Bio-Pharm Technology Co. Ltd. (Shanghai, China) (12).

Raw fastq files were demultiplexed, quality filtered by Trimmomatic, and merged by FLASH with the following criteria: (i) the reads were truncated at any site receiving an average quality score < 20 over a 50-bp sliding window; (ii) primers were exactly matched allowing two nucleotide mismatching, and reads containing ambiguous bases were removed; and (iii) sequences whose overlap was longer than 10 bp were merged according to their overlap sequence. The taxonomy of each 16S rRNA gene sequence was analyzed by the RDP Classifier algorithm (http://rdp.cme.msu.edu/) against the Silva (SSU123) 16S rRNA database using a confidence threshold of 70% (12).

Operational taxonomic units (OTUs) representing <0.005% of the population were removed. The relative abundance of each OTU was counted at different taxonomic levels. Then, bioinformatics analysis was mainly performed using QIIME (v1.7.0) and R packages (v3.2.0). The OTU table in QIIME was used to calculate OTU-level alpha diversity indices, while β-diversity was assessed by principal coordinate analysis (PCoA). Then, the composition and relative abundances of dominant bacterial taxa at the genus level were analyzed. LDA scores were computed to identify significant differences in the proportions of cecal microbiota.

### Metabolomics analysis of cecal contents

The extraction of cecal contents of metabolites and liquid chromatography-mass spectrometry (LC-MS) analysis were conducted based on previous studies (13, 14). The original data were preprocessed through the Majorbio platform, mainly including missing value recording and normalization. Briefly, the conversion of raw data, principal component analysis (PCA), and partial least squares-discriminate analysis (PLS-DA) were conducted. By comparing with the Human Metabolome Database (HMDB) 4.0 database, we obtained the classification information of metabolites in the differential metabolic set and performed statistical mapping. We also conducted one-way analysis of variance analysis (ANOVA) on the differential metabolites. The analysis was completed with the online platform of Majorbio ISanger Cloud platform (https://cloud.majorbio.com/).

### Network analysis among IMF content, cross-sectional area of LD muscle, microbiota, and metabolites

Furthermore, to clarify the relationship among IMF content, cross-sectional area of LD muscle, and the top 20 differential microbiota and metabolites, we conducted network analysis. The networks were built from correlation matrices. Each node shows one variable, and edges depict significant correlations. Modules were defined after Louvain clustering was applied to build networks.

### Statistical analysis

Comparison between the distinct groups was conducted using ANOVA using GraphPad Prism Version 9.4.1. Using non-parametric Spearman’s method, a correlation matrix was computed with every variable. To keep the correlation relevant, only those with an absolute rho coefficient above 0.6 and statistically significant (*P* < 0.05) were kept. The analysis was conducted utilizing the online platform of Majorbio ISanger Cloud (https://cloud.majorbio.com/) in conjunction with R packages. Statistical significance was denoted as follows:“*” means *P* < 0.05, “**” means *P* < 0.01, “***” means *P* < 0.001, “****” means *P* < 0.0001.

## RESULTS

### Myofiber properties of the LD at different stages of age

**Fig 1:**
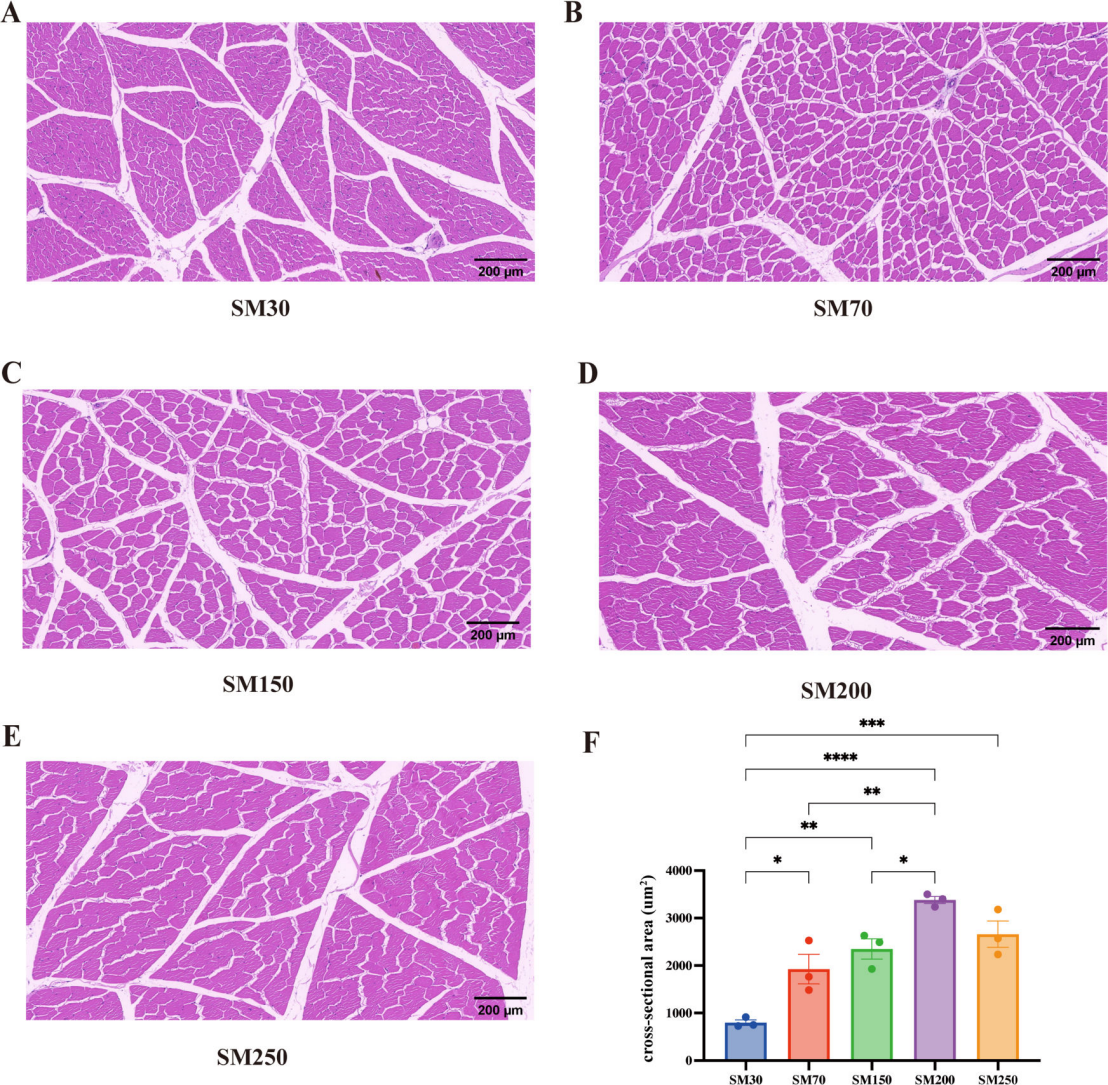
Comparison of NXP myofiber properties of LD among five ages (*n* = 3). (**A, B, C, D, and E**) Paraffin section of LD muscle (magnification: 100×). (F) Cross-sectional area of LD fibers. SM30, SM70, SM150, SM200, and SM250 represent NXPs at 30, 70, 150, 200, and 250 days of age. NXPs, Ningxiang pigs; LD, longissimus dorsi. Data were presented as the mean ± standard error of mean (S.E.M.). “*” means *P* < 0.05, “**” means *P* < 0.01, “***” means *P* < 0.001, and “****” means *P* < 0.0001.

The myofiber properties of the LD muscle in NXPs at different stages of age are compared in Fig. 1. The cross-sectional area of the LD muscle at 30 days of age was found to be significantly lower (*P* < 0.05) than that at 70, 150, 200, and 250 days of age. Moreover, at 200 days of age, the cross-sectional area of the LD muscle was significantly higher (*P* < 0.05) than that at 70 and 150 days of age.

### Fatty acids and intramuscular fat contents of LD muscle at different stages of age

**Fig 2:**
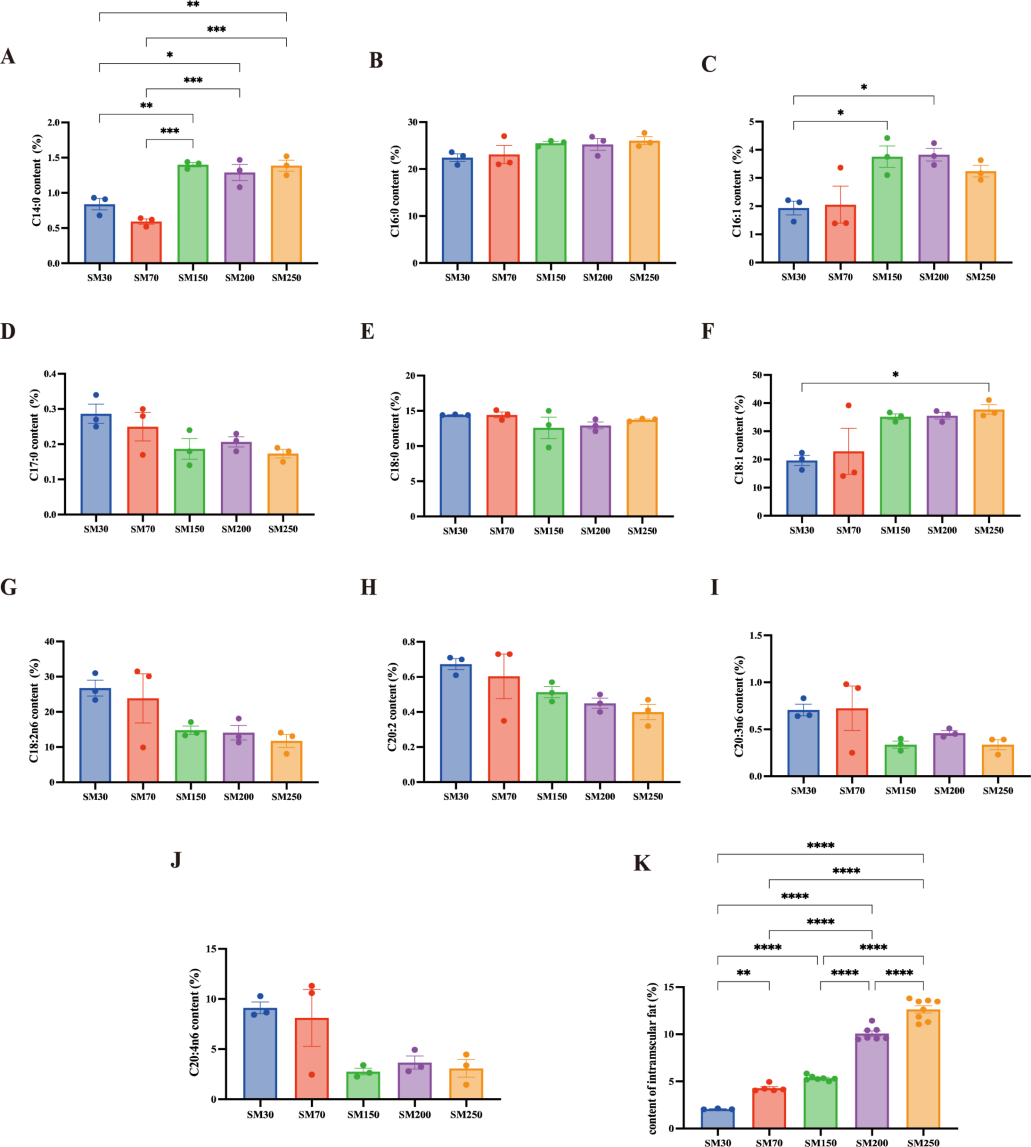
Comparison of the long-chain fatty acid and intramuscular fat content of LD in NXPs (*n* = 3). (**A**) C14:0 content, (**B**) C16:0 content, (**C**) C16:0 content, (**D**) C17:0 content, (**E**) C18:0 content, (**F**) C18:1 content, (**G**) C18:2n6 content, (**H**) C20:2 content, (**I**) C20:3n6 content, (**J**) C20:4n6 content, and (**K**) IMF content. SM30, SM70, SM150, SM200, and SM250 represent NXPs at 30, 70, 150, 200, and 250 days of age. NXPs: Ningxiang pigs. LD, longissimus dorsi; IMF, intramuscular fat. “*” means *P* < 0.05, “**” means *P* < 0.01, “***” means *P* < 0.001, and “****” means *P* < 0.001

The fatty acid and IMF contents of the LD muscle at different stages of age are presented in Fig. 2. The C14:0 content in the LD muscle at 30 and 70 days of age was significantly lower (*P* < 0.05) than that at 150 and 200 days of age. The C16:1 content at 150 and 200 days of age and the C18:1 content at 250 days were significantly higher (*P* < 0.05) than those at 30 days of age. Furthermore, the IMF content in NXPs increased significantly (*P* < 0.05) as the pigs’ age extended.

### Cecal microbiota profiling at different stages of age

**Fig 3:**
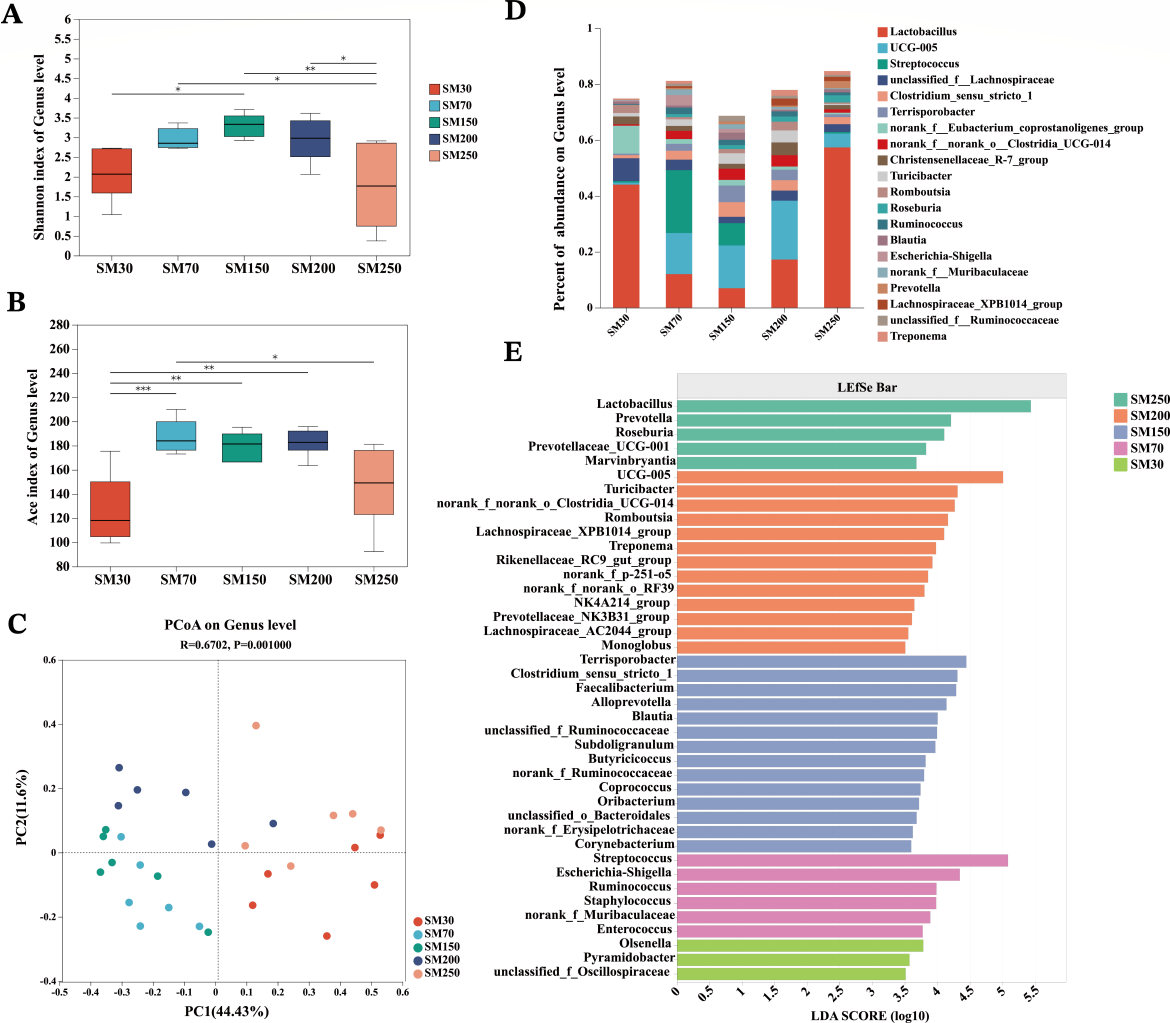
Comparison of cecal microbiota composition of NXPs (*n* = 6). (**A**) Shannon index of genus level. (**B**) Ace index of genus level. (**C**) PCoA on genus level. (**D**) Composition and relative abundances of dominant bacterial taxa at the genus level. (**E**) LDA scores computed for differences in the proportions of cecal microbiota. Taxa meeting an LDA significant threshold of >3.5 are shown. SM30, SM70, SM150, SM200, and SM250 represent NXPs at 30, 70, 150, 200, and 250 days of age. NXPs, Ningxiang pigs; ACE, abundance-based coverage estimator; PCoA, principal coordinates analysis; LDA, histogram of linear discriminant analysis. “*” means *P* < 0.05, “**” means *P* < 0.01, “***” means *P* < 0.001, and “****” means *P* < 0.0001

The composition of the cecal microbiota was analyzed using high-throughput sequencing of 16S rDNA. A total of 775,145,093 optimized sequences were obtained from 30 samples (*n* = 6 for each age stage), resulting in 1,141 distinct OTUs after double-ended sequence quality control splicing. To evaluate the diversity and abundance of the cecal microbial communities at different stages of age, alpha diversity analysis was conducted. The Shannon index of NXPs at 30 days of age was significantly lower (*P* < 0.05) than that at 150 days of age, and the Shannon index of NXPs at 250 days of age was significantly lower (*P* < 0.05) than that at 70, 150, and 200 days of age (Fig. 3A). The ACE index of NXPs at 30 days of age was significantly lower (*P* < 0.05) than that at 70, 150, and 200 days of age, and the ACE index of NXPs at 250 days of age was significantly lower (*P* < 0.05) than that at 70 days of age (Fig. 3B). These results indicated that cecal microbial diversity and abundance increased with age, reaching a peak at a certain age, and then begin to decline. Furthermore, beta diversity analysis revealed that, despite shared habitat conditions and diet, dots from different groups were distinctly separated. This highlights that the structure of the gut microbiota community is significantly influenced by age (Fig. 3C).

At the genus level, the relative abundances of microbiota varied among different stages of age, indicating the dominant bacteria’s dynamic changes in the NXPs during different stages of age (Fig. 3D). To further investigate the impact of age on gut microbial compositions, we used LEfSe analysis to distinguish the differential bacteria at the genus level. A total of 41 biomarkers were detected, with 3 bacterial genera at 30 days of age, 6 bacterial genera at 70 days of age, 14 bacterial genera at 150 days of age, 13 bacterial genera at 200 days of age, and 5 bacterial genera at 250 days of age. The analysis showed an enrichment of *Olsenella* at 30 days of age, *Streptococcus* at 70 days of age, *Terrisporobacter* at 150 days of age, UCG-005 at 200 days of age, and the genus *Lactobacillus* at 250 days of age in the NXPs (Fig. 3E).

### Cecal metabolite profiling at different stages of age

**Fig 4:**
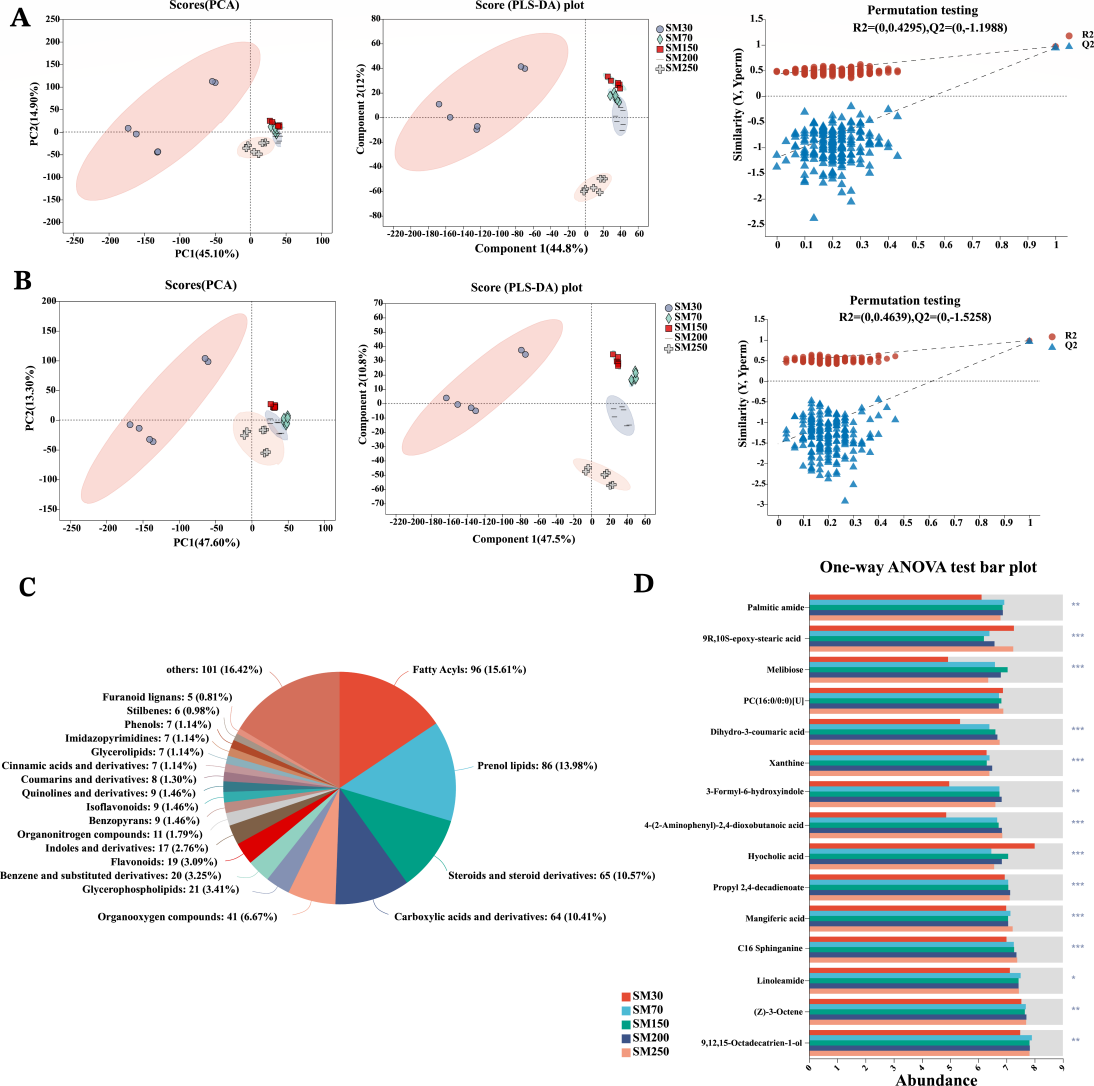
Comparison of cecal metabolome in NXPs (*n* = 6). (**A**) PCA plots in positive modes. (**B**) PCA plots in negative modes. (**C**) Pie chart based on counts of HMDB chemical taxonomy (“Class”) for differential metabolites. (**D**) Comparison of representative metabolites (top 15 ranked by abundance). SM30, SM70, SM150, SM200, and SM250 represent NXPs at 30, 70, 150, 200, and 250 days of age. NXPs, Ningxiang pigs; PCA, principal component analysis; HMDB, human metabolome database.

The cecal samples were subjected to LC-MS analysis in both positive and negative ion modes. Following peak alignment, 9,459 effective peaks and 496 differential metabolites were identified in the positive ion mode, and 9,418 effective peaks and 410 differential metabolites were identified in the negative ion mode. PCA and PLS-DA in the positive and negative modes revealed clear separation among different stages of age, which indicated significant differences in cecal metabolism in different groups (Fig. 4A and B). Seven hundred sixty-seven differential metabolites were assigned to the HMDB database, 615 of which were matched and classified into 21 HMDB classes. The classification of differential metabolites revealed that 15.61% of compounds were fatty acyls, 13.98% were prenol lipids, 10.57% were steroids and steroid derivatives, and 10.41% were carboxylic acids and derivatives (Fig. 4C). To investigate the differences in metabolite abundance, ANOVA analysis was employed to filter the metabolites. The top 15 metabolites are displayed according to their abundance ranking (Fig. 4D). Except for PC(16:0/0:0)[U], which demonstrated no significant difference, all other top 15 metabolites exhibited significant variations.

### Integrated networks of gut microbiota and metabolites associated with the IMF content and cross-sectional area of LD

**Fig 5:**
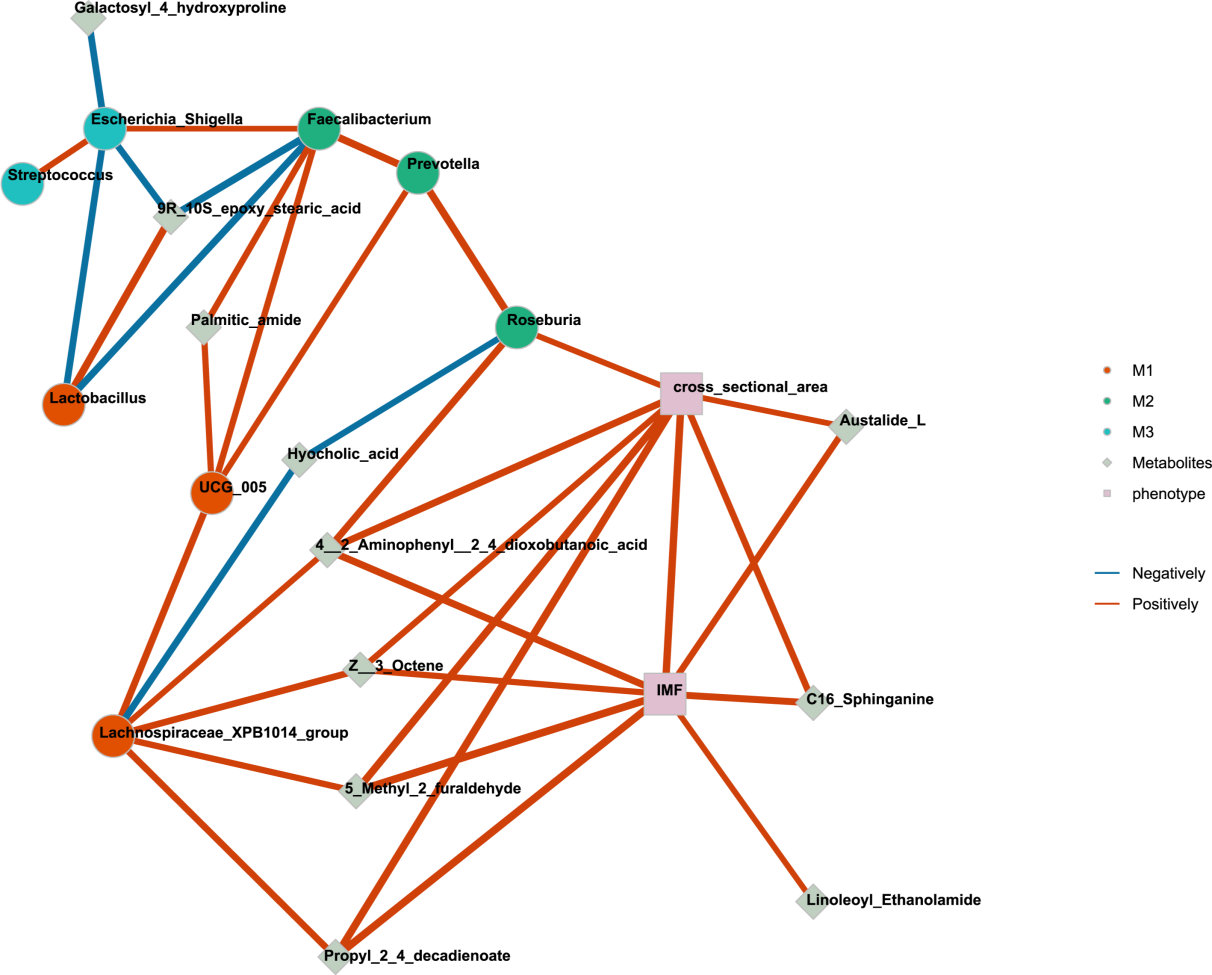
Integrated networks of gut microbiota and metabolites associated with the IMF content, cross-sectional area of LD. The network analysis among the top 20 differential microbiota and metabolites, IMF content, and cross-sectional area of LD muscle was conducted. The results are presented with a significance level of *P* < 0.05 and a correlation coefficient above 0.6. The red was positive correlation, and the blue was negative correlation. IMF, intramuscular fat; LD, longissimus dorsi.

The results of network analysis revealed a positive relationship between *Lachnospiraceae_XPB1014_group* and 4-2-Aminophenyl-2-4_dioxobutanoic-acid, (Z)-3-Octene, 5-Methyl-furaldehyde, Propyl-2-4-decadienoate. These metabolites were also positively correlated with IMF content and the cross-sectional area of LD. However, *Lachnospiraceae-XPB1014-group* showed a negative relationship with hyocholic acid, which, in turn, exhibited a negative correlation with *Roseburia*. On the other hand, *Roseburia* demonstrated a positive correlation with the cross-sectional area of LD (Fig. 5).

## DISCUSSION

Despite NXPs being a fat-type breed of pigs, there has been a paucity of research on the temporal changes in cecum microbiota and metabolism in this breed. Our study addresses this gap by revealing the dynamic shifts in cecal microbiota-metabolites during the post-weaning growth phase of NXPs. Additionally, we have established correlations between these dynamics and IMF deposition as well as the cross-sectional area development in the LD muscle, providing crucial insights into the metabolic and musculoskeletal aspects of NXPs’ post-weaning growth.

It has been shown that muscle fiber quantity and size undergo development before birth, with only the size of fibers increasing after birth. As body weight increases, the diameter and cross-sectional area of muscle fibers also increase (15). Our study found that the cross-sectional area of the LD muscle in NXPs gradually increased during their growth and development stages, peaking at 200 days of age. This finding is consistent with previous research on the subject (16). Some studies have revealed that a larger fiber diameter is indicative of a lower concentration of saturated fatty acids and a higher concentration of unsaturated fatty acids (17). However, this relationship was not identified in the current study, which could be attributed to factors such as the animal source and the number of experimental samples used.

Our results showed significant changes in cecal microbiota population of NXPs across growth stages. We observed that the microbial population initially increased, reached a plateau, and then declined. This result supports the observation of De Rodas et al. (18), who reported an increase in alpha diversity in various gastrointestinal tract (GIT) locations from birth to 84 days of age and decreased diversity in market samples (154 days of age) (18), In addition, Wang et al. (19) found that the microbial community diversity, as measured by the Shannon index, reached a plateau at 146 days of age (19), which is broadly consistent with our findings. This could suggest that the maturation of the gut microbiota in NXPs might occur at around 150–200 days of age. Following abundance and LEfSe analyses, we elucidated age-specific shifts in the dominant microbial taxa within NXPs. The observed shifts in microbial composition may be intricately linked to the varying physiological developments occurring at different stages (20). However, the specific relationships between microbial profiles and the nuanced aspects of physiological development remain to be thoroughly investigated.

Bacteria residing in the gut have the ability to produce various metabolites with specific biological functions, which play a crucial role in facilitating communication between the gut microbiota and host cells (21, 22). Additionally, these metabolites have the potential to influence other organs such as the liver, kidney, and brain through both portal and systemic circulations. This results in a more comprehensive regulation of host health and can even lead to systemic effects (21). Thus, we also conducted untargeted metabolomics to explore the shifts of intestinal metabolites in NXPs at different days of age. Metabolomic results showed that there were significant differences in the metabolite composition of cecal contents at different stages of age. Notably, the top two categories or classes in the HMDB chemical taxonomy for the identified differential metabolites were “fatty acyls” and “prenol lipids.” Fatty acyls and prenol lipids are two of the eight categories of lipids (23). They are common to all living organisms. The significant differences in lipid composition in the cecal contents of NXPs at different stages could be attributed to two factors: (i) digestive and absorptive capacity: NXPs may exhibit differences in their digestive and absorptive capacities at various growth stages, influencing the breakdown and absorption of lipids in the cecum (24) and (ii) microbial population: the microbial composition of the gut plays a crucial role in the degradation and metabolism of food. Variations in microbial types and abundance at different growth stages can impact the metabolism of lipids in the cecum (8). In the current study, the trends in the changes in alpha diversity of the microbial community align with the majority of differential metabolic trends. As age increases, there is an initial rise followed by a subsequent decline. This indicates that there is a certain relationship between the dynamic adjustment of the gut microbiota and the host’s metabolic state.

To elucidate the relationship, we proceeded with network analysis in the subsequent analysis. *Lachnospiraceae-XPB1014-group* and *Roseburia* are predominant genus within the butyrate-producing *Firmicutes* (25, 26). The increase of bacteria such as *Lachnospira* and *Roseburia* that produce butyrate was found to indirectly result in a decrease in bile salt concentration (27). This aligns with the negative correlation observed in this study between *Lachnospiraceae-XPB1014-group* and hyocholic acid, as well as between *Roseburia* and hyocholic acid. Moreover, four metabolites exhibited a positive correlation with IMF content, LD cross-sectional area, and *Lachnospiraceae-XPB1014-group*. (Z)-3-Octene belongs to the class of organic compounds known as unsaturated aliphatic hydrocarbons and may be involved in fatty acid metabolism. The metabolic pathway of Propyl-2-4-decadienoate belongs to fatty acid metabolism, which implies that Z-3-Octene and Propyl-2-4-decadienoate may play a role in the synthesis or accumulation of intramuscular fat. The metabolic pathways of 4-2-Aminophenyl-2-4-dioxobutanoic-acid belong to tryptophan metabolism. Tryptophan and its metabolites can influence various physiological processes, including muscle development and composition (28). 5-Methyl-furaldehyde can be obtained through the transformation of sugar compounds such as fructose, glucose, and cellulose and may also be derived from the feed processing process. This connection with IMF content and LD cross-sectional area suggests that dietary components or processing methods may contribute to muscle characteristics. The positive correlation with *Lachnospiraceae-XPB1014-group* suggests a potential microbial influence on the observed metabolites and muscle composition. Microbial activities in the gut may impact the metabolism of compounds and contribute to variations in IMF content and LD cross-sectional area. This is consistent with the results of previous studies (4, 29, 30). However, further exploration of the molecular interactions, gut microbiota composition, and specific pathways affected by these compounds can provide a more comprehensive understanding of the underlying mechanisms connecting these variables in the context of muscle metabolism and composition.

Previous research indicates that different pig breeds possess distinct gut microbiota and metabolic profiles, influenced by factors such as genetics, diet, and environment (31). Through our study and comparisons with other research, we found that as pigs grow older, the diversity of gut microbiota in almost all breeds increases gradually and then begins to decline after reaching a certain stage, with the peak age varying among breeds (18, 19). Moreover, our analysis revealed an enrichment of *Olsenella* at 30 days of age, *Streptococcus* at 70 days of age, *Terrisporobacter* at 150 days of age, UCG-005 at 200 days of age, and *Lactobacillus* at 250 days of age in the NXPs. These age-dependent shifts in the gut microbiome reflect a transition from primary lactate metabolism to an enhanced ability to metabolize plant polysaccharides (32). While the shift of functions was generally comparable to that in other breeds, detailed comparisons in terms of the abundance of genera and species, differences in the proportions of cecal microbiota at different ages, and metabolic functions are not possible due to the lack of comparative studies conducted under identical diets and environmental conditions. This represents a limitation of the current study and a key focus for future research.

### Conclusions

Our study has elucidated the dynamic changes of the cecal microbiota-metabolites during the post-weaning growth phase of NXPs. Furthermore, we have identified their correlation with IMF deposition and the development of the cross-sectional area in the LD muscle, which provides potential biological insights into age-based dynamic shifts of the gut microbiota’s microecological community and metabolites.
